# Retraction: Role of microRNA-15a-5p in the atherosclerotic inflammatory response and arterial injury improvement of diabetic by targeting FASN

**DOI:** 10.1042/BSR-2018-1852_RET

**Published:** 2021-08-24

**Keywords:** Arterial injury, Atherosclerosis, Atherosclerotic inflammation, Diabetes, miR-15a-5p

This Retraction follows an Expression of Concern relating to this article previously published by Portland Press.

This article is being retracted from *Bioscience Reports* at the request of the Editor-in-Chief and the Editorial Board following receipt of a notification from a reader, alerting the Editorial Board to highly similar bands in the Western blots of Figure 3B.

The authors were contacted regarding the concerns raised and provided the original scans of these Western blots, however given that the original scans show signs of manipulation, the Editorial Board stands by the decision to retract. The authors disagree to the Retraction.

